# The Development of Novel Drug Treatments for Stroke Patients: A Review

**DOI:** 10.3390/ijms23105796

**Published:** 2022-05-21

**Authors:** Dmitry Frank, Alexander Zlotnik, Matthew Boyko, Benjamin Fredrick Gruenbaum

**Affiliations:** 1 Department of Anesthesiology and Critical Care, Soroka University Medical Center, Ben-Gurion of the Negev, Beer-Sheva 84105, Israel; alexander.zlotnik.71@gmail.com (A.Z.); matthewboykoresearch@gmail.com (M.B.); 2 Department of Anesthesiology and Perioperative Medicine, Mayo Clinic, Jacksonville, FL 32224, USA; gruenbaum.benjamin@mayo.edu

**Keywords:** acute ischemic stroke, antithrombotic drugs, thrombolytic drugs, neuroprotection

## Abstract

Acute ischemic stroke is a critical condition that can result in disability and death. The consequences of this medical condition depend on various factors, including the size of the stroke, affected brain region, treatment onset, and the type of treatment. The primary objective of stroke treatment is to restart ischemic penumbra tissue perfusion and reduce infarct volume by sustaining blood flow. Recent research on the condition’s pathological pathways and processes has significantly improved treatment options beyond restoring perfusion. Many studies have concentrated on limiting injury severity via the manipulation of molecular mechanisms of ischemia, particularly in animal research. This article reviews completed and ongoing research on the development of acute ischemic stroke drugs. This study focuses on three main categories of antithrombotic drugs, thrombolytic drugs, and neuroprotective agents. The paper outlines findings from animal and clinical trials and explores the working mechanisms of these drugs.

## 1. Introduction

Stroke, also known as cerebral infarction, is a critical medical condition that compromises respiratory and cardiovascular function and causes severe neurological deficits. A considerable number of people experience recurrent or new stroke incidences each year. Stroke generally occurs through two mechanism, either ischemic, which is the most common, or hemorrhagic. Stroke is among the top five causes of death globally, especially in developed countries, and has a detrimental economic effect. These epidemiological and financial implications necessitate the development of effective treatment options, particularly for ischemic strokes. Although no existing medication consistently mitigates the effects of ischemic stroke, on-going drug research works to develop drugs to treat and prevent secondary complications. These drugs aim to support arterial recanalization, target metabolic and cellular metabolism, and modulate or inhibit the resultant inflammatory response.

Today, the standard treatments for stoke include aspirin regimens, recombinant tissue plasminogen activator (rtPA), and arterial recanalization technology. rtPA is the only FDA approved treatment for acute ischemic stroke and works through a process called thrombolysis, but only half of the patients on this medication recover fully [1]. A study by Gonzales et al. [2] indicated that the success rate was much lower at 35%. rtPA presents multiple contraindications, a modest success rate, and a restricted time window for its efficacy. There are safety concerns of using this method to treat patients who have undergone surgical procedures and those with other possible comorbidities [3]. Due to these inadequacies, research on the drug has adopted the approach of mixing it with other substances to improve its effectiveness.

Neuroprotection is one of the major foci of drug research. Initiatives in neuroprotective medication aim to minimize the destruction caused to the neuronal tissue during stroke [4]. Some of these drugs have shown promising results in animal tests but cannot replicate their effect during human clinical trials. This review will explore recent developments in new stroke medications to analyze their efficacy and highlight important factors to consider in future therapeutic modalities.

## 2. Etiology

**Figure 1:**
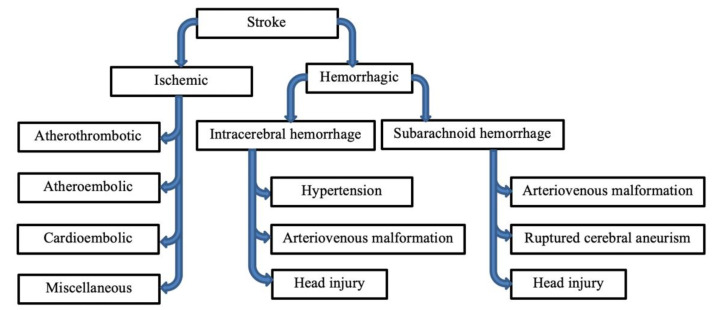
Classifications of stroke. Strokes are classified as either ischemic or hemorrhagic, which are further subcategorized.

Stroke is a multifactorial disease consisting of various pathologies and etiologies and requires precise diagnosis to differentiate the patient’s symptoms from other conditions that present similar symptoms [5]. Additionally, it must be determined if the type of stroke is hemorrhagic or ischemic [4]. Hemorrhagic stroke occurs when a patient suffers from ruptured intracranial vessels, resulting in inflammation and compression that cause neural death. Hemorrhagic stroke is divided into subarachnoid hemorrhage (SAH) and intracerebral hemorrhage (ICA). The main causes of SAH and ICA are arteriovenous malformation (AVM), ruptured cerebral aneurism (RCA), head injury and hypertension (HTN). Ischemic stroke can be divided into several subcategories: atherothrombotic, atheroembolic, cardioembolic, and miscellaneous (Figure 1).

Atherothrombotic stoke occurs in patients with comorbid and dyslipidemia pathologies such as diabetes mellitus and arterial hypertension. Arterial hypertension damages endothelium and smooth muscle function [4,6], while diabetes triggers macrovascular and microvascular angiopathy. The relationship between diabetes mellitus and the increased risk of stroke is multifactorial. Diabetes is associated with metabolic syndrome involving hyperglycemia, hypertension, and dyslipidemia. Even though the microvascular condition connected with hyperglycemia plays a significant role in the development of brain ischemia, scientists predominantly consider it a degenerative disease and therefore not a primary precipitant of acute ischemia. The conditions related to metabolic syndrome cause endothelial dysfunction, atheroma, and hypercoagulability, conditions that are all associated with stroke.

During atheroembolic stroke, a substance called atheromatous plaque builds up inside the ascending aorta or within the neck and head arteries, and tributaries contain reactive and damaged endothelial cells. In the Valsalva maneuver, a fracture of the friable thrombus occurs, secreting an embolus that moves upstream and embeds itself in the brain vasculature [5]. When the atheromatous plaque breaks apart, it secretes gelatinous cholesterol elements, which cause noticeable embolization inside brain arteries. This type of stroke happens mostly in patients with conditions associated with higher low-density lipoproteins and lower amounts of high-density lipoproteins. Atheroembolism manifests in small embolic particles lodged in arteries and may occur spontaneously or following arteriography, vascular surgery, or anticoagulation [7]. The clinical consequences of atheroembolism vary depending on the location of the target organ and the frequency and number of embolic episodes. Atheroembolism therefore has a wide variety of clinical presentations, from clinically silent attacks only recognized during diagnostic procedures to complex clinical procedures characterized by multiple organ involvement. The simultaneous or sequential involvement of various body segments may greatly facilitate a correct diagnosis in the case is subacute and subtle clinical presentation. Old age appears to be the most significant risk factor for atheroembolism when it occurs from an aortic source.

Cardioembolic stroke is the most common ischemic stroke and is clinically similar to atheroembolic stroke, although their etiologies are different [4]. It occurs when a clot formed inside the patient’s cardiac chambers detaches and travels peripherally, lodging inside small-caliber vessels and distally obstructing blood flow [5]. These emboli arise due to various pathologies, but most happen because of atrial fibrillation. Patients with atrial fibrillation typically have increased residence time of the fluid within their left atrium [8]. Coagulation and platelet aggregation may happen in atriums that are not sufficiently anticoagulated. Essentially, the cardioversion of the sinus rhythm in a patient with atrial fibrillation improves the amount ejected from the atria considerably, increasing the chances that a latent thrombus will detach and flow into the aorta. Given that the carotid arteries lie in the way, the embolus typically moves to the cerebral vasculature, obstructing blood flow to brain tissues and triggering an acute ischemic stroke. Cardioembolic stroke can also originate less frequently from emboli due to thrombi formed on diseased and prosthetic heart valves, vegetations due to systemic infections, and cardiac myxomas.

Miscellaneous causes of ischemic stroke include many thoracic or neck artery dissections, which cause the brain to lose perfusion. In addition, pulmonary thrombosis can obstruct blood flow toward the left side of the heart, causing brain ischemia. In addition, fat, air, or other non-thrombotic emboli account for a small fraction of acute ischemic stroke [5]. These miscellaneous causes, although less common than other forms of stroke, still occur frequently enough to warrant proper investigation of their causes and effects.

## 3. Pathogenesis of Stroke

**Figure 2:**
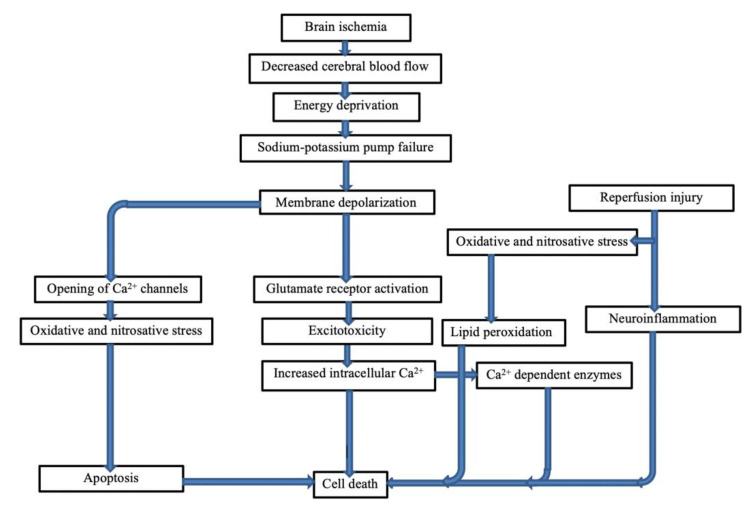
Ischemic stroke pathogenesis and possible targets of neuroprotection.

Stoke is characterized as a sudden neurological outburst resulting from impaired perfusion through blood vessels in the brain. Stroke can either be ischemic, the most common type, or hemorrhagic. In this review, we describe some of the important mechanisms of brain injury after stroke, including excitotoxicity, ionic imbalance, calcium dependent injury mechanisms, reperfusion, oxidative stress and nitrosative stress, and neuroinflammation (Figure 2).

### 3.1. Ischemic and Hemorrhagic Stroke

While ischemic strike results from a blocked brain blood vessel, hemorrhagic stroke happens when a brain blood vessel bursts, causing blood to leak into the brain. Both types of stroke result in loss of blood supply to the brain and, eventually, cell death [9]. As with all cells, neurons require glucose and oxygen, which is delivered through the blood, to survive and function. Millions of neurons could die when deprived of oxygen for a few minutes. Ischemia could also provoke edema, inflammation, and numerous other processes that that could continue damaging the brain tissue days following the initial insult. The most frequent cause of ischemic stroke is obstructive coagulation. Coagulation is a naturally occurring body function that proceeds from blood vessel trauma and is meant to reduce bleeding [10]. However, blood clots can also hamper normal blood flow, and induce a stroke. When a clot develops on the blood vessel wall and multiplies in size until it can impede blood flow, it is considered a thrombus. An embolus is a blood clot that breaks away from the blood vessel wall and moves along the blood stream. This can cause a cardio embolic stroke when the clotting originates from the heart. People with stenosis, atrial fibrillation, valve infections, and other heart conditions have a higher risk of developing this condition.

Chronic atherosclerosis, build-up of cellular debris and fatty deposits inside a blood vessel wall, is another condition with an associated risk of ischemic stroke. When atherosclerotic plaques develop, they trigger stenosis. Atherosclerosis could also activate cells that trigger clotting. Following an ischemic stroke, the brain contains ischemic penumbra. Reperfusion is critical to rescuing the damaged tissues and if this process is delayed, more penumbra tissues will die and result in an infarct.

Hemorrhagic stroke can either be subarachnoid or intracerebral [9]. An intracerebral hemorrhagic stroke manifests when a blood vessel in the brain ruptures and causes blood to leak into the brain tissue. Several conditions can make blood vessels in the brain susceptible to rupture, including amyloid angiopathy and chronic hypertension which can weaken the walls of these vessels. Blood thinning medicines and poor blood-clotting capabilities could enhance bleeding and result in stroke. Structural abnormalities in blood vessels during the brain development stage could also increase a person’s chances of developing hemorrhagic stroke. This type of stroke affects more females than males [11].

### 3.2. Excitotoxicity and Stroke

Several studies have indicated that glutamate excitotoxicity is the primary mechanism that triggers cell death in neurological conditions such as epilepsy, amyotrophic lateral sclerosis, traumatic brain injury (TBI), and stroke. Consequently, significant research is dedicated to establishing intervening mechanisms to control excitotoxicity and avert stroke damage. Research into glutamate excitotoxicity began when early studies established the neurotoxic characteristics of monosodium glutamate [12]. Studies into this compound, which is also used as a food additive, showed that it was neurotoxic to interior layers of mice retina. Subsequent investigations revealed that glutamate’s excitatory characteristics cause it to depolarize neurons, prompting them to respond. Researchers have since established that the neurotoxic effects of monosodium glutamate also affect central and peripheral neurons in rats, mice, rhesus monkeys, and rabbits [13].

Glutamate is a fundamental neurotransmitter, particularly in adult brains. Its primary function is to facilitate axon guidance, neuronal growth, brain maturation and development, and disease and health synaptic plasticity. Glutamate also plays a central role in facilitating neuron degeneration following stroke, typically as the primary neurotransmitter. Glutamate functioning occurs predominantly via activation of the N-methyl-D-aspartate (NMDA) metabotropic, an ionotropic glutamate receptor, to produce various signals according to the signaling proteins [14]. Synaptic activities prompt the NMDA receptor, resulting in the stimulation of crucial proteins. During an ischemia episode, especially a brief one, the extracellular glutamate levels rise sharply, stimulating NMDA receptors within extrasynaptic sites to cause excitotoxic neuronal death.

### 3.3. Excitotoxicity Mechanism

Several hypotheses have been proposed to explain the mechanisms of excitotoxicity. One accepted explanation is that the complex mechanisms of excitotoxicity are prompted by the activation of glutamate receptors that causes dendrites to degenerate and cells to die. This excitotoxic event affects all subcellular compartments and causes alterations in cell areas of the endoplasmic reticulum (ER), mitochondria, cytosol, and nucleus [15]. Normal levels of the glutamate activator can impair neurons when exposed to oxidative and metabolic stress, such as after traumatic brain injuries or stroke conditions. Neurodegenerative disorders associated with old age can also trigger this condition.

Excitotoxicity occurs when glutamate receptors become overactivated. This overactivation causes an influx of sodium and calcium cations all through the cellular plasma membrane, which causes the opening of NMDA and another glutamate receptor, AMPA; kainite-type glutamate receptors; and calcium cations, the metabolic part of calcium in the blood. Additionally, glutamate receptor activation stimulates the production of inositol triphosphate (IP3) and the production and secretion of calcium cations from ER [16]. The excitotoxicity varies according to the type of neurons involved; their state of development and environmental conditions; NMPA, AMPA, and kainite-type receptor contributions; metabotropic receptors; and channels of calcium cations. A neuron’s capacity to buffer and remove calcium cations is a critical determining factor of its excitotoxicity susceptibilities.

### 3.4. Ionic Imbalance

Electrolytes are crucial for proper bodily functioning due to their role in helping cells to transmit electrical impulses and maintain a voltage balance across their membranes. The magnesium, calcium, sodium, and potassium ions in the blood are the primary electrolytes charged with maintaining ionic and blood pressure balance [17]. Magnesium supports brain development and function. It also has a protective function in the brain as a glutamate receptor antagonist and as a calcium channel blocker. Calcium ions have a physiological function in multiple cerebral ischemia mechanisms. Calcium metabolism in cells during and shortly after a transient ischemia impacts the series of events that result in neural injury and death. Sodium regulates blood pressure, osmotic equilibrium, and blood volume and significantly assists in determining the acid-base balance. Potassium ions are essential for neural function, health, and cerebral function. Following an ischemic stroke, an ionic imbalance occurs, characterized by a decrease in potassium ions and an increase in sodium ions [18].

Ischemia results in reduced availability of glucose and oxygen to support aerobic production of ATP. Animal studies have shown that the levels of ATP production are depleted within three minutes of complete ischemia. This depletion triggers an increase in calcium ions which further speeds up ATP depletion [19]. There is little storage of either oxygen or glucose and ATP production during ischemia relies on aerobic glycolysis for as long as stores last. This results in continued ATP use, but suboptimal production of ATP to fuel aerobic metabolism, so a lactic acidosis develops. Loss of ATP causes failure of the membrane’s ionic pump function, resulting in efflux of potassium and influx of sodium, calcium, and chloride ions, marking the beginning of cytotoxic edema. The resulting ionic imbalance triggers a series of events that eventually cause neural death. The potassium leakage triggers cell depolarization, ion channel opening and the release of excitatory amino acids neurotransmitters such as aspartate and glutamate.

The excitatory amino acids cause further depolarization of neighboring cells and distal and uninfected regions of the brain that result in chloride and sodium influx via quisqualate and kainite receptors, with water exacerbating the intracellular edema. The excitatory amino acid also causes calcium influx via N-methyl-D-aspartate (NMDA) receptors and the release of excitatory transmitters. Excessive production of these NMDA receptors is a critical cause of ischemic injuries [20]. In addition, the excitatory amino acid causes a conversion of phosphorylases, uncoupling of oxidative phosphorylation, degradation of cytosolic proteases, activation of proteases, and lipase stimulation. The lipases liberate arachidonic acid and other free fatty acids that cause damage via the production of oxygen radicals and prostaglandins.

### 3.5. Calcium Homeostasis towards Cell Death

Calcium ions are crucial signaling molecules, and close regulation of calcium levels in cells is essential for proper cell function. Since calcium also facilitates glutamate-mediated injuries, any dysfunction of the cation’s homeostatic functioning has the ability to worsen calcium overload, promoting excitotoxicity [21]. Additionally, the initial influx of calcium cations following the stimulation of excitotoxic glutamate causes a surplus of calcium ions attributed to neural death. The sodium–calcium exchanger (NCX) found in the plasma membrane is a critical regulator of intercellular calcium levels that extrudes calcium via an influx of sodium ions [22]. The initial glutamate stimulation causes NCX to extrude calcium in order to restore intercellular calcium concentration to physiological levels. NMDA receptor-facilitated NCX dysfunction causes calcium overload after an excitotoxic stimulus which triggers a substitution of defective NCX with an isoform that blocks excitotoxic neural death [23]. NCX operates contrary to calcium extrusion during the early stages of glutamine stimulation since it occurs in the reverse direction.

Mitochondria are the other primary mechanisms in maintaining homeostasis of intercellular calcium. Mitochondria can restore normal calcium levels by absorbing a significant amount of the ions and enabling extrusion of ATP-dependent calcium. Thus, inhabitation of NCX and mitochondria prevents intercellular calcium recovery after glutamate stimulation. When this stimulation occurs, mitochondrial absorption of calcium prompts the production of reactive oxygen species (ROS), dilation of the mitochondrial permeability transition pore, and induction of calcium deregulation [24]. The opening of the pores causes depolarization of mitochondria. Some studies have suggested that the anti-diabetic medication metformin can demonstrate neuroprotective characteristics by hindering mitochondrial respiratory-chain-complex 1, enhancing the mitochondrial capacity for calcium and preventing the opening of its permeability pore [25]. Additionally, mitochondrial NCX facilitates calcium extrusion to the cytoplasm from the mitochondria, partially contributing to the deferred loading of cytoplasmic calcium during excitotoxicity.

The contribution of calcium ions to excitotoxicity explains the NMDA receptor’s predominant role in the process in comparison with glutamate receptors that are less permeable to calcium. It does not account for the neurotoxicity of NMDA receptor input, in contrast with other sources that produce neuroprotective calcium. For instance, voltage-gated calcium conduits encourage cultured neurons to survive in vitro and shield these cells from dying from NMDA receptor-mediated actions [26].

### 3.6. Activation of Calcium Dependent Enzymes

Increased calcium activates calcium-dependent enzymes such as proteases, endonucleases, phosphatases, and tissue transglutaminase [27]. Some of these enzymes are involved in cell death. In this regard, activation of calcineurin, a calcium-dependent phosphate, readily reduces cell death in the presence of low levels of serum. Moreover, the overexpression of a constitutively active form of calcineurin induces apoptosis without a calcium increase, implying that the activation of this phosphate is sufficient to trigger programmed cell death. Dephosphorylation of BAD, a pro-apoptotic protein of the BCL-2 family appears to explain the apoptogenic activity of calcineurin. In the absence of phosphate, BAD promotes caspase activation, apparently by overcoming the anti-apoptotic effect of BCL-X, at the level of Aaf-1 [28].

### 3.7. Mechanisms of Reperfusion Injury

Reperfusion damage occurs with critical clinical manifestations such as acute heart failure, myocardial hibernation, gastrointestinal dysfunction, cerebral dysfunction, multiple organ dysfunction syndrome, and systematic inflammatory response syndrome. Whenever this injury occurs, it must be controlled before it causes cell damage and organ failure. The obstruction of blood flow during ischemia can result in electron transport dysfunction in mitochondria. Reduced mitochondria production of ATP causes sodium–potassium pumps dysfunction, anaerobic metabolism, and ribosome detachment. The dysfunction of the sodium–potassium pump arises from the limited energy reaching the pump due to low ATP level [29]. The anaerobic metabolism results in reduced levels of anti-oxidative agents and ATP in cells. Lactic acid retention may cause metabolic acidosis.

Additionally, anaerobic metabolism may cause the calcium (Ca^2+^-ATPase pumps) and potassium–sodium pumps (Na^+^-K^+^-ATPase pumps) to fail on the surface of the cell. A higher sodium concentration in cells reduces the activity of Na^+^-H^+^ pumps. The Ca^2+^-ATPase pumps will also become dysfunctional, limiting the re-uptake of calcium. The build-up of calcium, hydrogen, and sodium ions results in hyperosmolarity. Hyperosmolarity forces water into the cytoplasm, causing cell swelling [30]. Hydrogen retention reduces cellular PH, impairing enzyme activity and nuclear chromatin clumping. In addition, ribosome detachment inhibits protein synthesis. Once the reperfusion stage is complete, blood is restored into the ischemic tissue to supply the tissues with oxygen.

Moreover, as a result, reactive oxygen species (ROS) regeneration increases because of the lower concentrations of anti-oxidative agents within ischemic cells. ROS triggers oxidative stress that encourages endothelial dysfunction, local inflammatory responses, and DNA damage. The resulting DNA damage adversely impacts DNA damage response [31]. The related oxidative stress and inflammatory responses may induce a cytokine disturbance that damages cellular structures causing cell death. This reperfusion stage occurs dynamically and may last several days.

Oxidative stress could result from enzymatic and non-enzymatic sources. Popular enzymatic sources include reduced nicotinamide adenine dinucleotide phosphate (NADPH) oxidase, xanthine oxidative, uncoupled nitric-oxide synthase and mitochondrial electoral transport chain systems. These enzymatic sources can be found in microphages, polymorphonuclear cells, and endothelial cells [32]. Meanwhile, non-enzymatic sources typically comprise oxidative stress sources such as myoglobin and hemoglobin, particularly during cases of severe ischemic injury. Increased oxidative stress and calcium overload can trigger apoptosis when the mitochondria are involved. The NADPH oxidase system, mitochondrial electron transport system and the xanthine oxidase systems participate in oxidative stress in multiple organs including the brain, heart, muscles, pancreas, kidney, stomach, lung, and intestines. Nitric oxide synthase is a primary stress element in aortic heart and liver endothelia cells.

### 3.8. Cell Damage during Ischemia–Reperfusion Injuries

Ischemic-reperfusion injuries can be described as the paradoxical worsening of cellular malfunction and death after blood is restored to cells that had earlier been affected by ischemic damage [33]. The sum of tissue injures attributed to ischemia–reperfusion injury generally falls under two groups: reperfusion injury ad ischemia injury. Ischemic injuries initially cause hypo-nutrition and hypoxia, but prolonged ischemia causes metabolic cell products to become retained and trigger metabolic acidosis. Once the blood supply is restored, the production of ROS rises, and local inflammation also increases, resulting in secondary injuries. Cell damage from prolonged ischemia–reperfusion injuries may case autophagy, necrosis, apoptosis, and necroptosis. Modest ischemia–reperfusion injuries may trigger cell dysfunction via autophagy and activate recovery mechanisms to promote survival. In cases where the ischemic damage is severe, necrotic, or apoptotic pathways may induce cell death. An ischemia–reperfusion injury occurring over a shorter duration may induce cell survival programs to manage cell damage and ROS generation. Several cell death mechanisms could be induced by multiple pathogenesis of ischemia–reperfusion injury.

### 3.9. Mechanisms of Cell Death Due to Ischemia–Reperfusion

Cell death is a crucial component of multiple medical conditions. Apoptosis is a form of cell death related to ischemia–reperfusion. It is a programmed process that occurs due to hypoxic stress during ischemic injuries following ROS production after reperfusion. Cell death via ischemia reperfusion can either be intrinsic or extrinsic [33]. Both pathways can influence and cross-communicate with each other. Death receptors and ligands, including the TWEAK, TNF-α, TRAIL, Fas ligard, and TL1A, activate the extrinsic pathway. It activates a caspase-8, protease, and cleave caspase-3 which cause cell death using the proteolysis mechanism in damaged cells. Hypoxia, cellular toxins, or radiation activate the intrinsic avenue, or mitochondrial pathway, and change mitochondrial membrane integrity, resulting in activation of pro-apoptotic BLC-2 family of proteins. During ischemic-reperfusion injuries, the cytoplasmic levels rise dangerously and bind the BCL-XL and BL-2. BCL-XL traditionally prevents the mitochondria from releasing cytochrome C, thereby protecting the body from the effects of ischemic injuries [34]. The sudden increase in BCL-XL and BL-2 occurs at the same time as the processing of BAK and BAX which are afterwards infused into the mitochondrial membrane to secrete pro-apoptotic proteins to activate pro-caspase-9. The azotosome causes a caspase cascade to support apoptosis through activation of caspace-9. The Endonuclease G also interacts with apoptosis-inducing factor resulting in DNA fragmentation. DNA damage causes PIDDosome formation by expressing P53-induced protein with a death domain (PIDD) and reacting with pro-caspase-2 and the caspase activation and recruitment domain (CARD) of receptor-interacting protein-associated ICH-1/CED-3 homologous protein with a death domain (RAIDD). The complex triggers caspace-2 to drive apoptosis. Nonetheless, apoptosis due to ischemic-reperfusion is not as frequent as necrosis.

Mitoptosis is another form of cell death, and it results from the fragmentation of mitochondria. During mitoptosis, BAK and BAX monitor mitochondrial cleavage and are associated with the outer mitochondrial membrane and dynamin-related protein 1 (Drp 1) instead of regulating cleavage proteins such as mitochondrial division 1 (Mdv1) and fission 1 (Fis 1). The regulation of mitochondrial cleavage and protein fusion results in fusion abnormalities and intercellular mitochondrial division.

Necrosis is similar to apoptosis since it is an execution-phase encoded cell death. Necrosis manifests via morphological changes such as cytoplasm vacuolation, disruption of the integrity of a cell membrane, cell content loss, and the secretion of proinflammatory molecules [35]. The necrosis form of cell death is characterized by early permeation of plasma membrane and organelle swelling. It occurs when cells undergo excessive external stress and is considered an unregulated and passive process. Although necrosis occurs in human pathology, few studies have explored its mechanism during ischemia–reperfusion injuries. Necrosis is triggered by a vivid alteration in the external environment prompted by biological, physical, and chemical damage. Organ swelling, cell damage, and mitochondrial functioning loss are the primary characteristics of necrosis, and the process prompts significant inflammatory response in ischemic tissues. In the past, studies showed that apoptosis occurs distinctly from necrosis. However, recent studies have demonstrated the necrosis occurs via specific molecular pathways [35].

### 3.10. Mechanisms of Oxidative Stress and Nitrosative Stress

Neuroinflammation and oxidative and nitrosative stress are acute pathological procedures during ischemia–reperfusion injuries which result in blood–brain barrier damage, neural damage, and hemorrhagic transformation following ischemic stroke. Increased production of nitric oxide may trigger further brain damage after ischemic stroke through enhancing oxidative stress and free radical formation. Oxidative and nitrosative stress is generally viewed as a condition in which cellular antioxidant defenses are insufficient to entirely incapacitate the reactive nitrogen species (RNS) and ROS due to the loss of defenses against oxidation and excessive ROS/RNS production and secretion. Excessive RNS and ROS production causes oxidative stress which damages tissues and cells while interfering with signaling pathways [36]. Oxidative and nitrosative stress also causes the mutilation of proteins, lipids, and nucleic acid bases. This damage can significantly compromise cell viability and health and prompt several cellular responses by generating secondary reactive species, eventually triggering cell death via apoptosis or necrosis.

The oxidative stress that causes ischemic cell death occurs via ROS formation. ROS is a collective term that describes peroxyl radial, lipid hydroperoxide, singlet oxygen, superoxide, hydroxyl radical, alkoxyl radical, ozone, and hypochlorous acid [37]. The oxidative stress process involves the overproduction of express free radicals which overwhelms the scavenging and detoxification capacity of cellular enzymes such as catalase, superoxide dismutase, and glutathione peroxidase. Non-enzymatic antioxidants such as glutathione, Vitamin C, and Vitamin E are also overwhelmed resulting in severe and rapid mutilation of lipids, DNA, and cellular proteins.

After cerebral reperfusion ischemic stroke, enhanced production of ROS/RNS occurs and causes neural injuries. The brain is highly vulnerable to assaults by free radicals due to its high lipid content. The reperfusion period results in elevated ROS levels in the cerebral vasculature and this could be an underlying trigger to post-ischemic-endothelial dysfunction. Several in vitro studies have shown that an increase in ROS levels damages several organs and tissues [38]. The NADPH oxidases are the only enzymes that have been associated with generation of ROS and superoxide within the cerebral vasculature.

These enzymes also secrete oxygen radicals. These enzymic conversations include cyclooxygenase-dependent transformation of arachidonic acid into prostanoids and hypoxanthine degradation, especially during reperfusion. This transformation occurs because cells are forced to rely on anaerobic glycolysis for energy during ischemic events [39]. Moreover, free radicals are also produced during the inflammatory reaction after an ischemic session. Xanthine oxidation and hypoxanthine oxidation is accompanied by hydrogen peroxide and generation of oxygen ions which intensify neural injuries during reperfusion. Oxidative and nitrosamine stresses are controlled via enzymes such as oxide synthase and superoxide dismutase (SOD).

Studies of mice with elevated SOD revealed reduced injury levels after a cerebral ischemic incident while those with less SOD enhanced injuries. Several studies have indicated that SOD reduces edema following ischemic injuries [40]. These findings reveal that the production of excessive oxygen radicals is important during ischemic brain injury. RNS/ROS prompt inflammatory responses which increase the secretion of pro inflammatory chemokines and cytokines. It also activates cytokine gene expression and leukocyte adhesion molecules by activating several transcription factors, including nuclear factor kappa-betta. Thus, ROS/RNS results in direct cell injuries and an increase in chemotaxis, leukocyte–endothelial adherence, and leukocyte activation after reperfusion. ROS/RNS causes considerable cellular destruction which ultimately cause tissue obliteration and cell death. These consequences result in protein denaturation, lipid peroxidation, cytoskeletal structure damage, chemotaxis, and DNA and nucleic acid damage.

### 3.11. Neuroinflammation

Neuroinflammation describes the brain’s response to inflammation, and it occurs following various acute brain conditions. Production of ROS, cytokines and chemokines regulates neuroinflammation by facilitating leukocytes [41]. Neuroinflammation causes the brain to release several cytokines, and the process also involves the reaction by several innate immune cells, or microglia, within the parenchyma, and infiltration by adaptive immune cells and myeloid cells. Neuroinflammation can occur after various types of brain injuries such as ischemic stroke [42]. However, proper functioning of the brain’s innate immune system depends on the astrocyte, glia cell expression, microglia, and interleukin release. Microglia and astrocytes are primary players in the multicellular response of the central nervous system to disease and trauma, which includes reactions by the immune system. Dysfunction in the metabolism of astrocytes and microglia prompt an accumulation of Aβ which triggers the secretion of neuroinflammatory mediators that encourage neurodegeneration [43]. The neuroinflammation process is determined by the duration, course, and circumstances of the neurological insult.

Thus, neuroinflammation can either be supportive or destructive depending on the immune indicators relayed to the central nervous system. The positive function of neuroinflammation only occurs briefly under controlled inflammatory situations and it manifests to protect the host organism. For instance, when low transient inflammation occurs, the immune cells will relay signals to the brain to increase interleukin-1 cytokine secretion which enhances the surveillance role of glia cells in the infected area of the brain. Expression of interleukin-4 promotes axonal regrowth and injury recovery. Meanwhile, the negative aspects of neuroinflammation primarily manifest as maladaptive responses to the inflammation. The characteristic features of this response include enhanced supraphysiological production of ROS, cytokines, nitric oxide synthases and other inflammatory mediators. The ROS assumes the role of a signaling molecule which mediates the inflammation [44]. These markers are evident in cases involving high trauma of the central nervous system and they enhance collateral damage. Acute phases of central nervous system trauma result in significant production of IL-1 and IL-6 which prompt a low-level inflammatory response, resulting in reduced neuronal plasticity and cognitive impairments.

## 4. Drug Research

**Figure 3:**
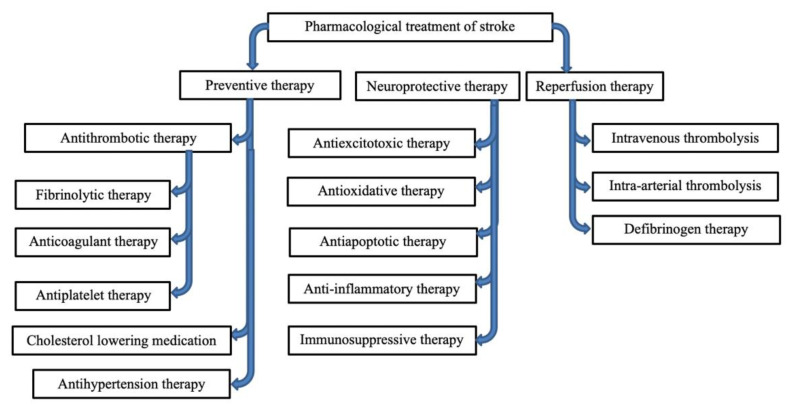
Summarized table of the pharmacological treatment of stroke.

The current approach for treating stroke concentrates on treating the acute phase. This approach slows down ischemia progress, then effects reperfusion and reescalation of brain parenchyma. Moreover, the treatment entails minimizing neuronal cell damage and death triggered by ischemia and the metabolic cascade caused by abrupt reperfusion [45]. The process involves using neuroprotective strategies and pharmacological approaches to slow down inflammatory feedback. Moreover, the treatment emphasizes retarding and rehabilitation of vascular disease progression to prevent additional strokes. A discussion of several pharmacological agents used in treating stroke provides an in-depth understanding of the drug development strategies. Generally, all pharmacologic treatments have three main objectives: prevention, neuroprotection, and reperfusion (Figure 3).

### 4.1. Thrombolytic

Most studies of stroke medication are based on models of ischemic stroke that analyze various mechanisms that occur during cerebral ischemic injuries. Insufficient oxygen levels trigger energy deprivation, and the arterial thromboembolic episode at the beginning is an ischemic cascade. The purpose of current trials on acute ischemic stroke is to liberate and reinstate ischemic penumbra within a particular treatment window. Otherwise, energy abrogation will deteriorate ion homeostasis and trigger an increase in the extracellular concentration of potassium ions and a reduction in chloride and sodium ions in the same region. The anoxic depolarization causes a glutamate release, development of reactive oxygen species, and dysregulation of calcium ions within the intercellular area. Seifert and Pennypacker [46] suggest that depolarization could cause a collapse of the mitochondrial membrane and neuroinflammation induction. Expeditious recanalization must occur to avoid an ischemic case that causes neuronal tissue infarction. Several drugs are undergoing tests for their capability to encourage neuroprotective pathways, alleviate ischemic injuries, degrade fibrin, and hinder platelet coagulation and aggregation. Neuroprotective agents can shield ischemic neurons during the acute phase of the stroke.

According to Balami et al. [47], thrombolysis can work against these mechanisms. Thrombolysis involves pharmacological drugs that cause a breakdown of clots to encourage blood flow. Thrombolysis is the standard treatment for stroke. The history of thrombotic drugs began in the 1960s and 1970s, when they were developed and underwent human trials. These drugs expedited clot formation to limit the extent of brain injury and encourage faster recovery after stroke [48]. The earliest studies on this treatment involved a comparison between a placebo and a population who received rtPA medication within three hours after experiencing a stroke [45]. The treatment group demonstrated significant improvements. However, the group receiving rtPA also experienced significantly higher levels of intracranial hemorrhage.

rtPA is a molecule of tPA genetically modified to act like endogenous tPA. The rtPA activates the zymogen plasminogen to produce an active plasmin enzyme [5]. The degradation of interlinked fibrin monomers causes the plasmin to generate soluble fibrin clots. Endogenous tPA traditionally occurs in small amounts, and it works by regulating the breakdown of fibrin plugs to maintain coagulation levels. The plasminogen activator inhibitor 1 (PAI-1) also contributes to tPA regulation through a reaction that hinders the degradation of the fibrin clot.

When rtPA is administered via infusion, the resultant insufficient PAI-1 levels cannot control tPA action, causing the body to have insufficient quantities to break down the fibrin clots [45]. The rtPA can induce platelet aggregation and binding of the fibrinogen of the platelets, without an increase in thromboxanes. Nonetheless, it is likely that adjunctive antiplatelet therapy with aspirin can prevent thrombosis after rtPA treatment. Some studies have revealed that rtPA activates platelets responsible for preventing aggregation [49]. However, there is general agreement that these treatments prevent the development of extensive platelet activation after tPA therapy. Many current guidelines continue to recommend rtPA thrombolysis as an intervention [50].

The FDA has only approved the alteplase-rtPA thrombotic to treat strokes. More recently, however, rtPA has declined in popularity for treating acute ischemic stroke due to its adverse effect on bleeding. This limitation has motivated researchers to search for additional thrombolytic agents that demonstrate a dependency on extensive fibrin selectivity without adversely affecting the central nervous system.

One recent approach involves the use of several treatments simultaneously. It has been observed that effective drugs contained a mixture of rtPA, lys-plasminogen-streprokinase, p-anisolylated, prourokinase, and urokinase [51]. Level II to V trials indicated the efficacy and safety of these medications when administered via intra-arterial and intravenous means [51]. Several supplementary techniques such as distal clot delivery, pulse spray delivery, mechanical clot disruption, and concomitant heparin and lys-plasminogen administration have also been studied for their efficacy [51]. However, studies have indicated that none of these techniques stands out yet as a gold standard.

Lee [52] showed that drugs such as microplasmin, plasmin tenecteplase desmoteplase, and reteplase have the potential to become viable thrombolytic agents. In particular, reteplase and tenectplase have garnered recent interest as possible treatment modalities. Reteplase, like alteplase, is a non-glyosylated tPA deletion mutein, but it has been modified to elongate its half-life and enhance its thrombolytic characteristics. Lyden et al. [53] performed a randomized feasibility study using a monkey model and found that the drug was viable for recanalization. In addition, an open-label and non-randomized test on acute ischemic stroke patients similarly observed the drug’s positive effects. The researchers administered reteplase three to six hours after the onset of the symptoms and noted improved recanalization rates when the researchers mixed abcximab and intra-arterial reteplase. Another study observed enhanced clinical outcomes when the drug was mixed with lyse fibrin to discourage platelet aggregation [54].

Similarly, the thrombolytic drug tenecteplase is also a genetically modified form of tPA. It has several advantages over alteplase, including higher fibrin levels, increased resistance to plasminogen, and a longer half-life. These advantages enable faster perfusion while minimizing the rate of symptomatic intracerebral hemorrhage incidence [55]. One study compared the performance of tenecteplase and alteplase when administered three to four hours after a stroke incidence [56]. These findings indicated that the patients on tenecteplase had a significantly higher reperfusion level than those of alteplase. A Scottish study also compared the two drugs but observed no significant differences in symptomatic intracerebral hemorrhage, salvaged penumbra, or other adverse impacts [57]. More research is necessary to establish the efficacy of these two drugs.

#### 4.1.1. Intravenous Thrombolysis (IVT)

IVT intervention using alteplase is the primary form of treatment for patients with ischemic stroke, as long as physicians begin the treatment within five minutes of symptom onset. IVT with alteplase has been shown to improve clinical results and revive inflammatory response in patients with cerebral ischemia [58]. The reason for the urgency is because the benefits of alteplase are highly time-dependent. Alteplase is a recombinant tPA and it works by initiating local fibrinolysis by binding fibrin within a thrombus and converting the entrapped plasminogen into plasmin. The resultant plasmin dissolves the thrombus.

#### 4.1.2. Mechanical Thrombectomy

Mechanical thrombectomy is suitable for patients with acute cases of ischemic stroke because it involves making a large arterial occlusion within the anterior circulation. In most cases, these patients undergo an IVT before they are treated using the mechanical thrombectomy procedure, a treatment called bridging therapy.

#### 4.1.3. IAT Intra-Arterial Thrombolysis Therapy

IAT intra-arterial thrombolysis therapy entails administering agents directly into an artery to dissolve a thrombosis. Several agents can be used for this procedure, but plasminogen activators are specifically utilized for intra-arterial thrombolysis. These activators encourage thrombolysis indirectly or direct via activating active plasminogen and plasmin to degenerate the fibrin within the blood clot. Nonetheless, IAT intra-arterial thrombolysis is yet to demonstrate clear clinical benefits for ischemic patients [59]. Thus, its effectiveness against ischemic stroke remains unclear.

#### 4.1.4. Fibrinogen Depleting Agents

Fibrinogen depleting agents are purified extracts from pit viper venom which can help ischemic stroke patients by cleaving circulating fibrinogen, resulting in reduced plasma viscosity and an increase in blood flow to affected areas [60]. The potential additional benefits include preventing the extension of clots and indirect thrombolytic effect by encouraging the release of endogenous tissue plasminogen activators from the endothelium. High fibrinogen levels can predict stroke recurrence in high-risk patients. The agent Ancrod acts on fibrinogen and inhibits the formation of cross-linked fibrin [61]. Omega-3 fatty acids, particularly eicosapentaenoic acid can also act as a fibrinogen depleting agent by reducing blood, lowering platelet aggregability and reducing viscosity through a decrease in fibrinogen levels.

### 4.2. Antithrombotic Drugs

Antithrombotic drugs consist of fibrinolytic, anticoagulants, and antiplatelets. Fibrinolytics dissolve clots, anticoagulants stop the formation of fibrin strands, and antiplatelets hinder platelet aggregation. These medications do not affect existing clots, unlike thrombolytics which dissolve existing ones, but instead aim to limit their growth.

An example of a recently developed antithrombotic drug is Eptifibatide, an antiplatelet medication under the glycoprotein inhibitor class. This drug works by binding onto glycoprotein IIb or IIIa between an activated platelet’s arms, effectively stopping the binding agent from fibrinogen and constraining thrombi formation. Researchers have combined the drug with heparin, aspirin, and intravenous rtPA treatments in varying doses to improve the shortfalls of rtPA medicine. Lippi et al. [62] observed that recent trials have investigated the drug’s safety standards and shown promising results. Similarly, Medcalf and Lawrence [63] experimented with mixing different dozes of rtPA and the tPA receptor annexin A2 and observed that the addition of A2 enhances the efficiency of rtPA in rats. Nevertheless, the rtPA medication proved insufficient in recanalizing extensive arterial injuries, hence the need for extensive research for alternative medicines.

### 4.3. Neuroprotective Therapy

Scientists have been studying neuroprotective medications for their potential to prevent partially damaged neurons from further injuries. These drugs seek to enhance functional recovery of damaged nerve cells in ischemic stroke patients. Essentially, they reduce stroke’s impact on brain tissues. Research on neuroprotective drugs is mostly focused on how these drugs can enhance neuronal healing and protect penumbral nerve ending from irreversible damage. Several neuroprotective agents exist, and these include glutamate receptors and related glutamate targets, magnesium sulfate, statins, melatonin, erythropoietin, and immunosuppressant drugs. Out of all the possible treatment modalities for stroke, neuroproteins have wide potential to provide an effective method for improving brain function (Table 1).

#### 4.3.1. Glutamate Receptors

Glutamine occurs throughout the human brain [64]. Given its potential impact on neural function, the body must closely regulate glutamatergic neurotransmission levels to avert over-activating the system. The brain involves different regulatory mechanisms to keep glutamatergic excitement within narrow boundaries. The control procedure involves glutaminase converting glutamine to glutamate [65]. The vesicular glutamine transporter then packs the glutamate into presynaptic vesicles before the neuron releases it via relations with SNARE proteins. The excitatory amino acid transporters in the glial cells then clear the glutamate from the extracellular area. The glutamine synthetase within the glial cells converts glutamate into glutamine. Various glutamate receptors are found on the postsynaptic and presynaptic neurons and on the glial cells, including metabotropic and ionotropic receptors [66]. Several factors determine the impact of the glutamate, including the postsynaptic density signaling proteins, localization, receptor subtype, and interactions with multiple signaling and scaffolding proteins.

Glutamate receptors are highly-probable candidates for the development of neuroprotective medication, due to the role of glutamate during the excitotoxic cascade. However, studies on glutamate receptors have produced ambiguous results. Some studies have shown that activated GluN2A with receptors has a beneficial effect. Specifically, the application of GluN2A with NVP-AAM077 and NMDA receptors could improve neuronal apoptosis induced by oxygen-glucose deprivation, exacerbate excitotoxicity induced by DL-three-betahydroxyaspartate or NMDA [67,68], and encourage ischemic damage flowing ischemia. Other studies have proposed that GluN2A reduced the extent of neural damage resulting from middle-cerebral artery occlusion [69]. Further research suggested that antagonizing GluN2A receptors with Zn^+^ or NVP-AAM077 reduces the excitotoxicity induced by NMDA in older hippocampal or cortical cultures [69]. A study by Yan et al. [70] showed that GluN2A that has been enhanced with tyrosine phosphorylation could be involved in the excitotoxicity process. However, the tyrosine kinases that demonstrated the highest potential in interrupting GluN2A phosphorylation and protecting CA1 neurons from ischemic processes are GluN2A and Cdk5. Sun et al. [71] studied the signaling pathway characteristics of GluN2A and noted that it plays different roles at varying times during cerebral ischemia. For instance, GluN2A encourages neural death in patients at the acute stage of stroke but induces neuronal survival afterwards.

It was also observed that GluN2B phosphorylation enhances cerebral ischemia and worsens ischemic brain injury [71]. When DAPK1 and Calcium/calmodulin-dependent protein kinases (CAMKII) bind to NMDAR glutamate receptor (GluN2B) near S1303, increased neural death due to ischemia takes place [72]. Tat-CN_21_ could minimize brain damage by stopping GluN2B from binding with CaMKII [73]. Thus, processes that hinder GluN2B phosphorylation could be a potential approach for treating ischemia.

#### 4.3.2. Other Glutamate Targets

Studies using other glutamate targets have shown some promising results in primates, rats, and human models. Milani et al. [74] showed that poly-arginine R18 is an appropriate candidate for neuroprotective agents for humans. They compared R18’s effectiveness to neuroprotective NA-1 peptide after administering the drugs intravenously in mice 60 min after middle cerebral artery occlusion (MCAO). R18 reduced the total infarct volume by 35.1% compared to 26.1% with NA-1. R18 also reduced the swelling in the hemisphere by 46.1% compared to 25.1% with NA-1 after 24 h. Essentially their study confirmed that R18 is a more viable neuroprotective agent than NA-1 and encouraged more research on the use of the drug in humans. NA-1 and R18 possess anti-excitotoxicity characteristics, inhibit proprotein convertases, and reduce calcium influx.

Animal studies have also established that blood glutamate scavengers can reduce subarachnoid hemorrhage caused by stroke and brain injuries. There are four blood glutamate scavengers: pyruvate, oxaloacetate, glutamate-oxaloacetate transaminase, and glutamate-pyruvate transaminase. Boyko et al. [75] studied the effect of pyruvate and oxaloacetate injected intravenously and demonstrated its glutamate blocking and reduction abilities. Their research established a significant reduction in glutamate concentrations 24 h after administration. It also showed that pyruvate reduced cerebrospinal fluid glutamate while oxaloacetate caused neurological performance. Additionally, both drugs penetrated the BBB, thereby demonstrating their effectiveness as neuroprotective drugs. Gruenbaum et al. [76] also demonstrated the efficacy of pyruvate and oxaloacetate glutamate scavengers in reducing blood glutamate levels in animal studies. An earlier study by Boyko et al. [77] showed that low levels of pyruvate did not result in any neuroprotective properties. When they administered a dose of 250mg/kg pyruvate in rats with MCAO, however, the researchers observed reduced brain edema and small infection volume reductions. Frank et al. [78] confirmed pyruvate’s neuroprotection properties and noted that the drug reduces anxiety-related behavior in rats. A mixture of glutamate-pyruvate transaminase (GBT) and pyruvate displayed similar neuroprotective abilities, attesting to the viability of both drugs in treating ischemic stroke.

#### 4.3.3. Magnesium Sulfate

The human body has an abundant amount of magnesium cations. Magnesium facilitates a variety of physiological activities and has many clinical uses, including regulating the calcium availability in cells, activating enzymatic processes involving transforming ADP to ATP, blocking NMDA receptors, and enabling neuroprotection and analgesia. A reduction in magnesium in the body results in hypokalemia and hypocalcemia [79]. Magnesium sulfate also possesses neuroprotective and anti-excitotoxicity properties, has the ability to impede NMDA, and can regulate calcium channels.

Magnesium can potentially protect glia and neurons from ischemic damage via several models. It reduces cerebral infarct volume due to its impact on neuronal actions, brain blood flow, or both. Following a central cerebral artery inclusion, the blood flow at the core falls below 10 mL/100 g/min [80]. The neurochemical and metabolic consequences of ischemia spark a chain reaction that, unless slowed down or stopped by reperfusion via neuroprotective drugs, will cause the penumbra’s death. These processes include extreme release by neurotransmitters, especially glutamate, and excessive postsynaptic receptor activation, including NMDA. Additionally, they trigger excessive calcium and sodium ion absorptions by cells via voltage and ligand channels and the initiation of calcium-dependent enzyme systems that trigger the production of free radicals, proteolysis, breakdown of membrane lipids, inflammation response, and apoptosis. Magnesium interferes with these processes by suppressing them [81]. Its ions are antiexcitotoxic since they inhibit glutamate release following an ischemia event. They also suppress the NMDA ion receptor channel while providing a voltage-dependent ion block. Animal models have shown that intraperitoneal, intravenous, and oral magnesium administration can penetrate the brain, ameliorate NMDA-induced brain injury, prevent subsequent seizures, and support neural action mechanisms [81].

Animal models have shown the benefits of magnesium following forebrain ischemic injuries, focal head injuries, spinal cord ischemia, and subarachnoid hemorrhage [82]. It enhances functional and behavioral outcomes following spinal cord ischemia or head injury and prevents seizures triggered by various electrical or chemical stimuli. Magnesium sulfate consistently subdues cerebral infarct volume following occlusion of the middle cerebral artery in rats.

Human studies have also shown magnesium’s neuroprotective action of modulating glutamate action. Kirkland et al. [83] observed that magnesium appears to improve a patient’s neurological functioning for more than 30 days after stroke. Trials have confirmed the benefits of paramedics administering magnesium to stroke patients before reaching the hospital [84]. Magnesium facilitates faster diagnosis and treatment [84]. Additionally, multiple studies have documented the use of magnesium to treat aneurysmal subarachnoid hemorrhage [85], TBI [86], and acute stroke [87]. However, magnesium sulfate in intensive care unit (ICU) patients is associated with a reduction in several biomarkers. Patients in the ICU who are prone to high mortality due to low serum magnesium must have their magnesium levels closely monitored.

#### 4.3.4. Statins

Physicians frequently administer statins to lower cholesterol levels. Statins have also shown efficacy in reducing cardiovascular events and coronary plaque [88], and have anti-inflammatory and antioxidant properties [89]. Simvastatatin, mevastatin, rovovastatin, and atorvastatin have demonstrated their anti-inflammatory and antioxidant abilities in animals and human subjects [90]. Statins work via multiple mechanisms, including reducing oxidized LDL and malondialdehyde and synthesizing endothelial nitric oxide type III. They also alter inflammatory gene expression in molecules such as ICAM-1, VCAM-1, interleukins, and E-selection. Studies on using statin to treat acute ischemic stroke patients have generated varying results. Some statins have improved cell penetration due to their lipophilic properties [91]. Meanwhile, other studies have associated statins with an increased risk of developing symptomatic hemorrhagic transformation due to these drugs’ fibrinolytic and antithrombotic properties.

#### 4.3.5. Melatonin

Apart from regulating circadian rhythm, melatonin, also known as N-acetyl-5-methoxy tryptamine, has multiple clinical applications. The pineal gland naturally produces melatonin, but scientists can also manufacture it in laboratories. Melatonin is a potent free radical scavenger and antioxidant that ameliorates neuroinflammation and accelerates brain tissue renovation. Several pre-clinical animal studies and clinical tests on stroke patients have investigated melatonin’s therapeutic properties. In vitro and in vitro studies have demonstrated that melatonin protects glial cells and encourages functional recovery, enhanced behavioral outcomes, and diminished inflammatory response in ischemic animals [92]. Lee et al. [93] study showed that melatonin has no impact on lateral striatum damage, suggesting that motor protection normalization only requires cortex protection.

Another study by Shinozuka et al. [94] showed that melatonin effectively shields cells from secondary death, impacting functional deficits. Nevertheless, most studies have confirmed that pre-treatment reduces cerebral infarction. Evidence indicates that melatonin treatments encourage enhanced glial cell survival, sustain homeostasis of the neural cell membrane, and help envelop action in cells by sucking excess potassium. It helps the cell’s water handling capacity. Melatonin works as a neuroprotective agent by altering the expression of antioxidant enzyme genes such as glutathione peroxidase, catalase, and superoxide dismutase. It also attenuates activator protein 1 (AP-1) and nuclear factor-Kappa B (NF-kB) downregulates cyclooxygenase 2, tumor necrosis factor-alpha, and interleukin-1-betta [95]. Melatonin decreases phospho-Jun N-terminal Kinase 1, leading to apoptotic factors suppression [96]. Additionally, it directly detoxifies free radicals and protects DNA.

#### 4.3.6. Erythropoietin

Erythropoietin is a naturally occurring hormone and cytokine in the human body. It is produced in the liver and kidneys. Erythropoietin promotes erythroid progenitor cell proliferation, survival, and differentiation. Recently, its non-hematopoietic properties have attracted the attention of new research. In particular, studies have documented its protection of the brain, kidney, heart, and retina cells against ischemic injuries [97]. Experiments using mid-gestation mice models showed that the neural tube epithelium, where proliferating neuron precursors are based, expresses high erythropoietin levels. In humans, the cortex, hippocampus, and midbrain produce the most erythropoietin. 

Studies with animal models have demonstrated the neuroprotective properties of erythropoietin in the central nervous system. The erythropoietin receptor (EpoR) excretes the erythropoietin that produces the neuroprotective effect in humans. Erythropoietin works by inhibiting apoptosis. It stimulates the Janus tyrosine kinase by signaling pathways that result in the expression of extracellular-regulated kinase, Bcl-2 [98], nuclear factor-kappa, and protein kinase. Researchers consider it a viable candidate for treating neurodegenerative conditions, including ischemic stroke, because its molecules are small enough to cross the BBB easily. Nevertheless, clinical tests have revealed adverse side effects including tumor growth and erythropoiesis.

#### 4.3.7. Sodium Channel Blockers

Sodium channel blockers work by inhibiting the influx of sodium via cell membranes. This blockade slows down the amplitude and rate of preliminary rapid depolarization, minimizes conduction velocity, and reduces cell excitability. Sodium channel blockers fall into the category of antiarrhythmic class 1 together with beta blockers in class II, potassium channel blockers in class III and calcium channel blocker in class IV. Sodium channel blockers have been used by physicians to treat chronic pain and were originally developed as antiarrhythmic and anticonvulsants [99]. Although these blockers have benefited some patients, their narrow therapeutic index limits their benefits.

#### 4.3.8. Lubeluzole

Lubeluzole is a form of benzothiazole that has demonstrated neuroprotective features in experiments involving nitric oxide synthesis, inhibition of glutamate release and blockage of calcium and sodium ion chilling [100]. Lubeluzole is also a S-enantomer benzothiazol compound with neuroprotective action in stroke. As a non-NMDA receptor antagonist, it inhibits accumulation of extracellular glutamate while normalizing the excitability of neurons. This compound does not interfere directly with NOS but attenuates glutamate-activated NOS pathways. It also inhibits voltage-activated sodium and calcium ion channels, though these channels are less potent and lack stereo specificity compared to other methods. When lubeluzole is administered within six hours after induction of infarct in stroke models, lubeluzole neutralizes neural damage and lesion volume [101]. Thus, this drug has shown significant promise in treating ischemic stroke patients.

#### 4.3.9. Mexiletine

Mexiletine is an antiarrhythmic and treats pain, comparable to an oral analogue of lidocaine. Pain physicians may provide intravenous lidocaine for pain management, with monitoring of dose and effect. After receiving an intravenously-administered drug, treatment many be readily converted to oral mexiletine. Mexiletine can also be used as a treatment for thalamic stroke pain, diabetic neuropathy, myotonia and allodynia [102].

#### 4.3.10. Calcium Channel Blockers and Chelators

Most stroke incidents occur because of obstructed blood flow in large and small arteries. This obstruction causes an influx of calcium ions into the brain, resulting in cell death. Calcium blockers slow down brain cell damage and death by preventing calcium ions influx. DP-b99 is a membrane activated calcium chelator currently in phase II acute stroke trials. If intracellular calcium accumulation contributes to neurotoxicity, then cell-permeant calcium chelators may be neuroprotective. It acts as a neuroprotective agent when it selectively modulates intercellular iron, zinc, and calcium [103].

#### 4.3.11. GABA (Gamma Aminobutyric Acid Agonists)

GABA is a receptor agonist that helps protect the brain against cases of acute stroke. Examples of GABA drugs include clomethiasole and diazepam. Clomethiasole and diazepam are GABA agonists that potentiate the activity of GABA, the main inhibitory neurotransmitter in the brain [104]. The drugs are mainly used as a sedative and anticonvulsant. Clomethiazole can be used as a GABA to decrease excitatory neuro-transmission by increasing activity of inhibitory pathways. The drug has proven effective in global and focal cerebral ischemia. In a preclinical evaluation of focal ischemia in rats, clomethiazole was administered one hour after reperfusion and tissue preservation was observed [105]. However, some studies have indicated that GABA has some associated side effects for individuals with acute stroke.

#### 4.3.12. Antioxidants

Naturally occurring antioxidants such as vitamins E and C are believed to reduce cell damage by quenching the dangerous free radicals that result from oxygen processing in the body. Antioxidants, both synthetic and natural, have demonstrated their potential for treating stroke since they can be administered after an ischemic stroke to minimize cell damage.

#### 4.3.13. NXY-059

A study by Diener et al., confirmed the potential of the agent NXY-059 in trapping free radicals and serving as a neuroprotectant [106]. Their randomized, double blind, and placebo-controlled trials on ischemic stroke patients gave intravenous infusion of NXY-059 within six hours before the onset of stroke symptoms. Their experiment also showed that the antioxidant prevents alteplase-associated hemorrhage.

#### 4.3.14. AEOL-10150

AEOL-10150 is a wide-spectrum metalloporphyrin SOD that is particularly designed to defuse reactive nitrogen and oxygen species. AEOL-10150 is a catalytic antioxidant molecule that is able to scavenge a wide range of ROS, or free radicals. As a catalytic anti-oxidant, AEOL-10150 copies and enhances the body’s natural response system against these damaging compounds. AEOL-10150 is believed to be a useful remedy for amyotrophic lateral sclerosis and has shown significant promise in radiation therapy and mustard gas exposure therapy [107]. AEOL-10150 has shown impressive results in animal studies and has demonstrated its safety for human use as a catalytic anti-oxidant drug.

#### 4.3.15. Deferoxamine

Deferoxamine is an iron chelator and an FDA-approved drug for treating acute iron overload and chronic iron intoxication resulting from transfusion-dependent anemia. Deferoxamine can penetrate the BBB rapidly and accumulate within the brain tissue following systemic administration. Deferoxamine chelates iron by establishing a stable complex that bars iron from participating in more chemical reactions. In vivo, deferoxamine can minimize hematoma and hemoglobin–induced edema. Some studies have demonstrated that deferoxamine reduces ICH induced neural death, neurological deficits, and brain atrophy [108]. Deferoxamine works by binding ferric iron and preventing hydroxyl radical formation via the Fenton/Haber–Weiss reaction. Deferoxamine reduces hemoglobin-induced brain K^+^/Na^+^ ATPase inhibition and neural toxicity. Favorable effects of iron chelator therapy have been reported in multiple ischemia models [109]. Deferoxamine also act as a free radical scavenger which can induce ischemic tolerance in the brain. However, the compound may cause hypersensitivity reactions, hypertension, and weight loss.

#### 4.3.16. Pyruvate

Pyruvate has many mechanisms of neuroprotection. It is a potential scavenger of ROS and can act as an antioxidant during brain ischemia. Pyruvate therapy in high doses has been suggested for stroke treatment because pyruvate provides rapid uptake via monocarboxylate transporters, and the body can utilize it immediately in mitochondria. Pyruvate prevents ischemia, edema and calcium overload, and acidosis while helping to preserve organs against action potential. When an organ is flooded with a secondary solution containing pyruvate and alcohol to stop functioning and vasodilate the vessels to facilitate full vascular flow, the pyruvate will preserve the organ’s energy state. However, mitochondria still remain the primary energy source of cell energy and these need significant amounts of oxygen to function as required. During ischemia, mitochondrial icotinamide adenine dinucleotide (NAD) + hydrogen (H) accumulates, resulting in the accumulation of cytosolic NADH and conversion of cystosolic pyruvate into lactate [110]. The NADH converts dihydroxycetone phosphate into glycerol phosphate so that the latter product also accumulates during ischemia.

Extracellular glutamate during ischemic stroke can sometimes become abnormally high. Gluamate transporters within capillarity endothelial cells can help reduce glutamate levels. However, the efficient of this efflux relies on the gradient of the glutamate concentration between the interstitial fluid and the blood. The body can lower glutamate content by activating the glutamate-pyruvate transaminase enzyme [111]. The pyruvate converts the glutamate into 2-ketoglutarate, reducing the concentration of glutamate in the blood.

It is known that ischemic injury induces overactivation of poly-ADP ribose polymerase-1 (PARP) that induces cell energy deficit by depletion of cytoplasmic NAD+. Nevertheless, overactivation of PARP lead to inhibition of hexokinase [112]. Pyruvate is capable of attenuating cell death in the astrocyte-neuron culture [113]. Pyruvate has also shown promising results in animal studies as a potential treatment for inflammation and stroke [114].

#### 4.3.17. C-Jun N Terminal Kinase (JNK) Inhibitor

JNK is a critical kinase that the brain secretes in response to stress, such as from multiple brain insults. Several studies have indicated that JNK- arbitrated signaling pathways help in relieving myocardial and cerebral reperfusion injury. JNK is among the stress response molecules that encourage the mitochondrion to engage in apoptosis signaling. One study demonstrated that IQ-IS effect on brain tissue was manifested as JNK-increase attenuation in rat hippocampus following focal ischemia [115]. It established that administering 20 mg/kg/IP/day of SP600125 for seven days considerably suppressed p-JNK expression levels, indicating possible reversal effects. Immuno-fluorescent findings reinforced Western blot findings that ischemia enhanced p-JNK phosphorylation, while SP600125 minimized its immuno-reactivity. Khan et al., examined JNK phosphorylation on the ipsilateral side of hippocampus and cortex through Western blot [116]. They established that chronic ischemia caused p-JNK phosphorylation in hippocampus and ipsilateral cortex of adult mice.

#### 4.3.18. Immunosuppressant Drugs

Tacrolimus and cyclosporine are immunosuppressants with neuroprotective properties [117]. Researchers have used these drugs extensively in animal studies and have demonstrated their ability to bind to immunophilins, blocking calcineurin and suppressing cells and interleukin-2 production [118]. Studies have also noted their neuroprotective mechanisms of blocking extracellular signal-regulated kinases (ERK 1/2) [119]. Ischemic state triggers ERK 1/2 production, thereby causing pro-apoptotic properties. Tacrolimus inhibits nitric oxide and calcineurin production and reduces tumor-necrosis factor-alpha.

#### 4.3.19. Antihypertension Therapy

Hypertension is a significant risk factor for stroke. ICH patients often have higher blood pressure levels. Almost a third of the patients with ICH experience hematoma expansion lasting more than twenty hours. Hematoma expansion and the initial hematoma volume are potent mortality indicators following an ICH incidence.

Pharmacological anti-hypertension treatments focus on preventing significant cardiovascular complications including stroke. Anti-hypertensive drugs typically work by controlling, preventing, and treating hypertension [120]. Hypertensive drugs that have shown the potential for treating ischemic stroke patients fall under the categories of beta blockers, diuretics, calcium channel blockers, and angiotensin-converting enzyme inhibiters [121]. These anti-hypertensive drug classes give different results concerning enhancing patient quality of life, lowering their blood pressure or left ventricular mass regression.

## 5. Conclusions

In conclusion, further research is needed to create a drug that can effectively treat stroke. Although studies have explored a wide range of drugs, the main categories of potential treatment modalities include antithrombotic, thrombolytic, and neuroprotective drugs. While the FDA has approved a therapy to treat systems associated with acute ischemic stroke, especially thrombolytics, its side effects prevent widespread use. Current studies work to determine the impact of stroke and the drugs that could effectively treat these symptoms. Here, we emphasize the potential of neuroprotective therapies, which so far have been understudied in their potential to treat stroke symptoms and other effects of brain injury. Although studies have explored a wide range of drugs, the main categories to emerge from this research include antithrombotic drugs, thrombolytic drugs, and neuroproteins. Neuroproteins have demonstrated significant potential to relieve stroke symptoms through considerable research. We anticipate that more research on neuroprotection will elucidate these options.

## Figures and Tables

**Table 1:** Table of Mechanisms of Action of Neuroprotectors.

Neuroprotectors	Mechanism of Action
NV-AAMO77	Antiexcitotoxic, antagonizes GluN2A receptors
Zn^+^	Antiexcitotoxic, antagonizes GluN2A receptors
Tat-CN_21_	Antiexcitotoxic, minimizes brain damage by stopping GluN2B from binding with CaMKII
Poly-arginine R18	Antiexcitotoxic, inhibits proprotein convertases, reducing calcium ion influx
NA-1	Antiexcitotoxic, inhibits proprotein convertases, reducing calcium ion influx
MgSO_4_	Antiexcitotoxic, noncompetitive NMDA_R_ blocking, regulation of calcium channels
Simvastatin, Mevastatin, Rovostatin, Atorvastatin	Anti-inflammatory, reduces oxidized LDL and malondialdehyde and synthesizes endothelial nitric oxide type III, alters inflammatory gene expression in molecules such as ICAM-1, VCAM-1, interleukins, and E-selection
Melatonin	Antioxidant; antiapoptotic; alters the expression of antioxidant enzyme genes such as glutathione peroxidase, catalase, and superoxide dismutase; attenuates AP-1 and NF-kB downregulating cyclooxygenase 2, tumour necrosis factor-alpha, and interleukin-1-B; protects cells from dying by inhibiting JKN 1 and suppressing apoptotic factors; detoxes free radicals and protects DNA
IQ-IS	Antiapoptotic, C-Jun N Terminal Kinase Inhibiting
SP600125	Antiapoptotic, C-Jun N Terminal Kinase Inhibiting
AEOL 10150	Antioxidant
Deferoxamine	Antioxidant
NXY 059	Antioxidant
Glutamate-oxaloacetate transaminase	Antiexcitotoxic. blood glutamate scavenging
Glutamate-pyruvate transaminase	Antiexcitotoxic. blood glutamate scavenging
Oxaloacetate	Antiexcitotoxic. blood glutamate scavenging
Pyruvate	Antiexcitotoxic, antioxidant, blood glutamate scavenging, anti-inflammatory
Erythropoietin	Antiapoptotic. stimulates the Janus tyrosine kinase by signalling pathways, expressing of extracellular-regulated kinase, Bcl-2, nuclear factor-kappa, and protein kinase
Lubeluzole	Sodium channel blocking
Mexiletine	Sodium channel blocking
DP b99	Calcium channel blocking
Clomethiasole	Gamma aminobutyric acid agonist
Diazepam	Gamma aminobutyric acid agonist
Cyclosporin	Immunosuppressing, antiapoptotic, suppresses cells and interleukin-2 production, blocks ERK 1/2
Tacrolimus	Immunosuppressant, antiapoptotic, suppresses cells and interleukin-2 production, blocks ERK 1/2, inhibits nitric oxide and calcineurin production, reduces tumour-necrosis factor-alpha
Metformin	Antioxidant, antiapoptotic, anti-inflammatory, hinders mitochondrial respiratory-chain-complex 1, enhances the mitochondria’s calcium capacity and prevents the opening of its permeability pore

## Data Availability

Not applicable.
